# *Staphylococcus aureus* Lipoprotein Induces Skin Inflammation, Accompanied with IFN-γ-Producing T Cell Accumulation through Dermal Dendritic Cells

**DOI:** 10.3390/pathogens7030064

**Published:** 2018-07-29

**Authors:** Suguru Saito, Ali F. Quadery

**Affiliations:** 1 Division of Clinical Nephrology and Rheumatology, Kidney Research Center, Niigata University Graduate School of Medical and Dental Sciences, Niigata 9518510, Japan; 2 Institute of Bio Medical Science, Academia Sinica, Taipei 115, Taiwan; 3 Biofluid Biomarker Center, Niigata University, Niigata 9502181, Japan; all-bbc@ccr.niigata-u.ac.jp

**Keywords:** *S. aureus*, lipoprotein, skin inflammation, dendritic cell, effector T cell

## Abstract

*Staphylococcus aureus* (*S. aureus*) is a commensal bacteria on the human skin, which causes serious skin inflammation. Several immune cells, especially effector T cells (Teff), have been identified as key players in *S. aureus*-derived skin inflammation. However, the bacterial component that induces dramatic host immune responses on the skin has not been well characterized. Here, we report that *S. aureus* lipoprotein (SA-LP) was recognized by the host immune system as a strong antigen, so this response induced severe skin inflammation. SA-LP activated dendritic cells (DCs), and this activation led to Teff accumulation on the inflamed skin in the murine intradermal (ID) injection model. The skin-accumulated Teff pool was established by IFN-ɤ-producing CD4^+^ and CD8^+^T (Th1 and Tc1). SA-LP activated dermal DC (DDC) in a dominant manner, so that these DCs were presumed to possess the strong responsibility of SA-LP-specific Teff generation in the skin-draining lymph nodes (dLN). SA-LP activated DC transfer into the mice ear, which showed similar inflammation, accompanied with Th1 and Tc1 accumulation on the skin. Thus, we revealed that SA-LP has a strong potential ability to establish skin inflammation through the DC-Teff axis. This finding provides novel insights not only for therapy, but also for the prevention of *S. aureus*-derived skin inflammation.

## 1. Introduction

Commensal bacteria, resident on the human skin in the order of trillions, has two different aspects such as maintaining host skin immunity and generating inflammation on the skin [1]. As a beneficial effect, they establish immune balance and defense for our skin. One of the commensal bacteria strains, *Staphylococcus epidermis* promotes IL-17A^+^CD8^+^T cell accumulation on the host epidermis, so that it establishes a natural defense against other pathogens such as *C. albicans* [2]. However, several commensal bacterial strains induce severe inflammation on the skin [3]. The underlying mechanism of the generation of skin inflammation is still unclear in several parts; however, the inflammation may progress and become a serious situation without any proper treatment. Therefore, commensal bacteria-originated skin inflammation has been recognized as a critical factor that should be avoided not only in clinical situations, but also in daily life.

*Staphylococcus aureus* is a well-known pathogen of serious skin inflammation, and the bacteria frequently colonizes on the inflamed skin. Atopic dermatitis (AD) is a well-known skin inflammation characterized by abnormal *S. aureus* colonization [4]. In the inflamed site on the skin, several subsets of T cell accumulation are usually observed, together with other immune cells recruitment [5]. For instance, IFN-ɤ^+^, IL-4^+^, or IL-17A^+^CD4^+^T cells (Th1, Th2, or Th17) and IL-17A^+^ɤδT cells, are famous effector T cells (Teff) that increase in atopic dermatitis (AD) skin [6,7]. These cells are orchestrated to establish the total immune response for the generation of skin inflammation. On healthy skin, commensal bacteria does not induce an inflammatory response at all without critical basic disease-like immunodeficiency, as our immune system allows for their residence as a symbiotic effect [8]. When the skin has been injured by factors such as mechanical and chemical damage, especially invading the epidermis layer deeply, it will be a trigger to induce a strong inflammatory response against the commensal bacteria, because abundant immune cells acquire the opportunity to interact with the bacteria [9]. In fact, it is well known that epithelial barrier disruption promotes AD or AD-like symptoms bearing a large amount of *S. aureus* colonization [10].

Even though several host side critical factors related to initiating/promoting commensal bacteria-originated skin inflammation has been understood, the key factor which has a dominant role in activating the host immune system on the bacterial side is still controversial. *S. aureus* is composed by a rigid outer cell wall containing several components such as peptidoglycan (PGN), lipoteichoic acid (LTA), wall teichoic acid (WTA), and cell wall/membrane proteins including lipoproteins [11,12,13,14,15]. These are located on the outer layer of the bacteria, so that these components potentially possess the chance to frequently interact with the host immune cell. From this concept, several studies have targeted these cell wall components, and have revealed the mechanism behind the induction of an inflammatory response in the host immune system by PGN and LTA through the Toll-like receptor 2 (TLR2), and activating the inflammatory cytokine production in immune cells [16,17]. However, these antigenic functions have not simply been adapted to the pathology of skin inflammation; therefore, any convincing findings have been reported for the pathogenesis of these cell wall components in skin inflammation. This evidence provides an insight into other cell wall components that strongly activate the host immune system at not only the single cell level, but also on the whole immunological network in the tissue.

Lipoprotein is a cell wall protein in Gram-positive bacteria including *S. aureus*, and has various functions for bacterial survival on the host skin [15]. In one of the *S. aureus* strains, USA300, around 70 lipoproteins have been identified with their predicted microbiological functions [15]. The predictable function in the majority of these lipoproteins have been determined from its structure; however, the exact functions and roles of these components are still unknown. Even though the *S. aureus* lipoprotein has many unclear parts, the immunological role of the immune cell has been elucidated mainly by using in vitro studies. This is the same as other potential antigens derived from *S. aureus*, lipoprotein through Toll-like receptor 2 (TLR2), which subsequently activates the Nuclear Factor-*kappa* B (NF-*k*B) pathway to induce inflammatory cytokine production [18,19]. In addition to the evidence, one study used a systemic *S. aureus* infection animal model and showed that lipoprotein recognition by the host immune system was indispensable for the establishment of the total inflammatory response [20]. From these points, bacterial lipoprotein is expected to be a potentially strong inflammation inducer on the skin.

Without any dependency for each character of the antigens derived from commensal bacteria including *S. aureus*, the substances must be recognized by the skin dendritic cells (DCs) in one of the initial steps to activate the host immune response [21]. Due to having a strong recognition and phagocytic ability against an ectopical substance, skin DCs capture various substances through a receptor-mediated manner [22]. The incorporated substance is processed as an antigen in general, so that it is presented to naive T cells through antigen presenting molecules such as the major histocompatibility complex (MHC) for priming, which is an important mechanism for antigen-specific Teff generation [23]. There are indispensable steps for the establishment of inflammatory responses based on Teff accumulation in infected/inflamed sites on the skin; hence it must be adopted for understanding the pathogenesis for *S. aureus*-originated skin inflammation.

In this study, we revealed that *S. aureus* lipoprotein (SA-LP) induced strong inflammation on the skin with the activation of the DC-Teff axis. We confirmed that SA-LP-activated DCs in a TLR2 dominant manner. The SA-LP activated skin DCs, which migrate into the skin-draining lymph nodes (dLNs), and SA-LP was processed as an antigen for priming naive T cells. In addition to these fundamental findings, a SA-LP intradermal (ID) injection into the ear exhibited dramatic thickness in the murine model. The inflamed skin possessed abundant IFN-γ-producing CD4^+^ and CD8^+^T cells (Th1 and Tc1) in the model. The underling mechanism of Teff accumulation was regulated by the dermal DC (DDC), which migrated from the skin into dLN as it specifically reacted to SA-LP. Furthermore, SA-LP-stimulated DC transfer into the mouse ear showed a similarly inflamed condition on the skin as the SA-LP ID-injected mice, and the inflamed skin possessed Th1 and Tc1 accumulation. This is the first report to address the evidence of *S. aureus*-derived lipoprotein inducing serious skin inflammation. These findings will form a valuable direction towards an understanding of the novel mechanisms in *S. aureus*-related skin inflammation.

## 2. Results

### 2.1. *S. aureus* Lipoprotein Activates Dendritic Cells through TLR2

To investigate the potential antigenicity of the *S. aureus* lipoprotein, we extracted crude lipoprotein (SA-LP) from MRSA, one of the *S. aureus* strains. The extracted SA-LP was purified by dialysis, then separated by SDS-PAGE and visualized with silver stain to confirm the quality. The purified SA-LP showed several clear bands in a range of around 10–60 kDa (Figure S1A). These patterns were similar to previous reports [18,19,24], hence we were able to confirm that the extraction and purification quality were sufficient. Previous studies indicated that each fraction of SA-LP separated in a size-dependent manner showed different activation status in the immune cell [18,19]; therefore, we investigated the activity of size-dependent fractionated lipoprotein (L1–4) in mouse bone marrow derived dendritic cell (BMDC) stimulation. Unexpectedly, all of the lipoprotein fractions activated the BMDCs by inducing abundant TNF-α production (Figure S1B). However, these were significantly lower than the stimulation by SA-LP. We concluded that *S. aureus* lipoprotein had an absolute antigenicity for DC, while it was not dependent on the size specificity. Thus, we decided to use SA-LP as a strong antigen throughout this study.

**Figure 1:**
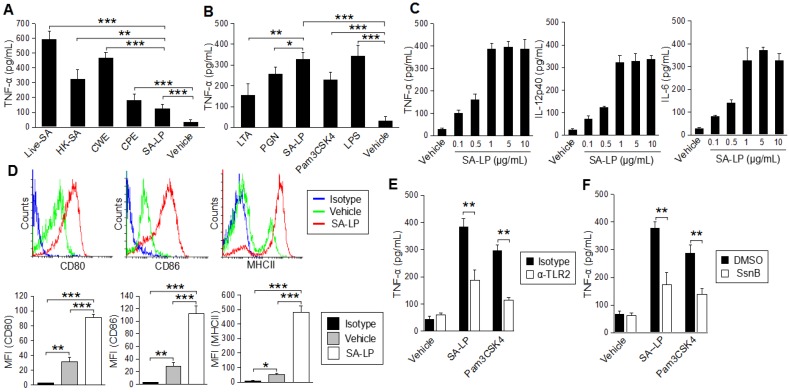
SA-LP activates DCs in a TLR2-dominant manner. (**A**,**B**) BMDCs (1.0 × 10^6^) were stimulated with the *S. aureus* derived component or whole bacteria. Cultured medium was harvested after 24 hr of stimulation. The TNF-α production was measured by ELISA. (**A**) Live-SA (10^6^), HK-SA (10^6^), CWE, CPE, SA-LP (extracted from 10^6^ of *S. aureus*). (**B**) LTA (10 μg/mL), PGN (10 μg/mL), SA-LP (10 μg/mL), Pam3CSK4 (500 ng/mL), LPS (100 ng/mL). (**C**) BMDCs (1.0 × 10^6^) were stimulated with SA-LP for 24 hr. Cultured medium was harvested, and the production of TNF-α, IL-12p40, and IL-6 were measured by ELISA. (**D**) Activation marker of SA-LP-stimulated BMDCs. BMDCs were stimulated by SA-LP (1 μg/mL) for 24 hr, then the stimulated cells were analyzed by flow cytometry to detect CD80, CD86, and MHCII. (**E**,**F**) BMDC stimulation under the blocking of TLR2 signaling. TLR2 was blocked with anti-mTLR2 mAd (10 μg/mL) (**E**), or inhibited with Sparstolonin B (100 μM) (**F**). The BMDCs were stimulated with SA-LP (1 μg/mL) for 24 h. Cultured medium was harvested and the TNF-α production was measured by ELISA. Live-SA; Live-*S. aureus*, HK-SA; Heat-Killed *S. aureus*, CPE; crude protein extract, CWE; cell wall extract, LTA; lipoteichoic acid, PGN; peptidoglycan, SA-LP; *S. aureus* crude lipoprotein. Data are shown as the mean and SD of at least three samples of a single experiment and are representative of at least three independent experiments. The Student’s *t*-test was used to analyze data for significant differences. Values of * *p* < 0.05, ** *p* < 0.01 and *** *p* < 0.001 were regarded as significant.

For further characterization of SA-LP in the immune response on DC, we performed BMDC stimulation by SA-LP, and compared their activity with other cell wall components derived from *S. aureus*. In the stimulation with whole bacteria (10^6^ of Live-SA and HK-SA), the BMDCs were strongly activated, and the cells produced abundant TNF-α. In addition to these results, CWE (originating from 10^6^ of *S. aureus*) also possessed strong antigenicity for BMDCs. In the stimulation of CPE and SA-LP (originating from 10^6^ of *S. aureus*), these substances showed less TNF-α production from the stimulated cells when compared with stimulation by whole bacteria, however, these were significantly higher than the vehicle control (Figure 1A). We also performed BMDC stimulation with an identical amount of cell wall components derived from *S. aureus*. In this assay, SA-LP showed a strong ability for BMDC stimulation when compared with other well-known cell wall components such as LTA and PGN. SA-LP-induced TNF-α production was significantly higher than these components in the BMDCs (Figure 1B). Thus, we concluded that the *S. aureus* originated lipoprotein had an absolutely strong antigenicity for DC.

To evaluate the activated status in SA-LP-stimulated BMDCs in detail, we investigated other inflammatory cytokine production and surface marker expressions. Not only TNF-α, but also IL-12p40 and IL-6 production were promoted by SA-LP in a dose-dependent manner (Figure 1C). The MHCII class II and costimulatory molecules, CD80 and CD86, were all up-regulated by SA-LP stimulation (Figure 1D). Taken together, SA-LP activated BMDCs and promoted abundant inflammatory cytokine production.

*S. aureus* lipoprotein action through TLR2 in a dominant manner has already been reported [18,19,25]. To investigate whether SA-LP could also be recognized by TLR2 on DCs, we performed BMDC stimulation by using SA-LP together with the blocking and inhibition of TLR2. The BMDCs treated with anti-TLR2 monoclonal antibody (mAb) showed significantly less TNF-α production when compared with that without blocking (isotype control antibody) (Figure 1E). TLR2 inhibition by using Sparstolonin B (SsnB), a TLR2 antagonist derived from a Chinese herb [26], also significantly attenuated TNF-α production from SA-LP-stimulated BMDCs (Figure 1F). Thus, SA-LP was recognized by a TLR2-dominant manner, and it activated inflammatory cytokine production on DCs similarly to other immune cells.

### 2.2. *S. aureus* Lipoprotein Promotes Dendritic Cell Migration

**Figure 2:**
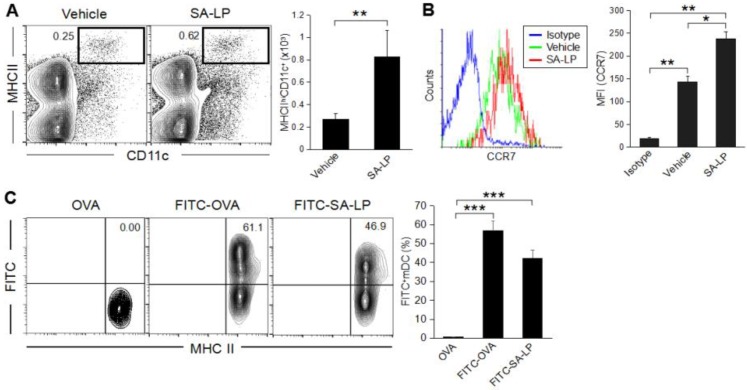
Characterization of migratory DCs in the murine skin inflammation model. (**A**–**C**) Wild type (WT mice were treated with SA-LP (10 μg) or vehicle solution (0.9% saline) by ID injection into the ear. After 48 hr of the treatment, skin-dLN were extracted from the mice, then isolated cells were used for flow cytometry analysis. (**A**) The population of mDC (MHCII^hi^CD11c^+^) in skin-dLN. The percentage of mDC was calculated from the flow cytometry image. (**B**) CCR7 expression on mDC. The histogram indicates CCR7 expression detected in the population of mDC (MHCII^hi^CD11c^+^). The MFI of CCR7 was calculated from the histogram. (**C**) In vivo antigen tracking in SA-LP ID mice. FITC-labeled SA-LP (10 μg), FITC-OVA (10 μg, positive control), or non-labeled OVA (10 μg, negative control) was applied by ID injection into the WT mice ear. After 48 hr of the treatment, skin-dLNs were extracted from the mice, then isolated cells were used for flow cytometry analysis. The plot indicates the cells gated on MHCII^hi^CD11c^+^ population. FITC^+^mDCs were characterized as MHCII^hi^FITC^+^. The MFI of FITC in mDC was calculated from the flow cytometry image. Data are shown as the mean and SD of at least three samples of a single experiment and are representative of at least three independent experiments. The Student’s *t*-test was used to analyze data for significant differences. Values of * *p* < 0.05, ** *p* < 0.01 and *** *p* < 0.001 were regarded as significant.

To investigate the immune response on the skin after exposure to SA-LP, we established a murine skin inflammation model by intradermal (ID) injection into the ear due to evidence that topical application of commensal bacteria on the healthy skin does not induce an inflammatory response, while ID injection induces a strong inflammatory response [2]. It is suspected that commensal bacteria itself or a bacterial component needs to penetrate the epidermal layer and reach the dermis to induce a severe inflammatory response on the skin. SA-LP ID-injected mice showed a high number of migratory DCs (mDCs), which were characterized as MHC class II^hi^ CD11c^+^ in the skin-draining lymph node (dLN) when compared with the vehicle control (Figure 2A). It is well known that DC migration to skin-dLN is regulated by chemokine receptor 7 (CCR7) [27]. With reference to the evidence, CCR7 expression was upregulated on mDC in SA-LP-treated mice (Figure 2B). To track the transported SA-LP as an antigen into skin-dLN via mDC, we performed in vivo antigen-tracking using the FITC-labeling method [28]. The population of mDCs in the skin-dLN possessed an absolutely high percentage of FITC-positive cells (MHCII^hi^CH11c^+^FITC^+^) in both the OVA-FITC- and SA-LP-FITC ID-injected mice when compared with the non-labeled OVA (Figure 2C). Taken together, SA-LP promoted DC migration from the skin to dLN, which infers the transport of SA-LP as an antigen.

### 2.3. SA-LP Induce Skin Inflammation Accompanied with T Cell Accumulation

**Figure 3:**
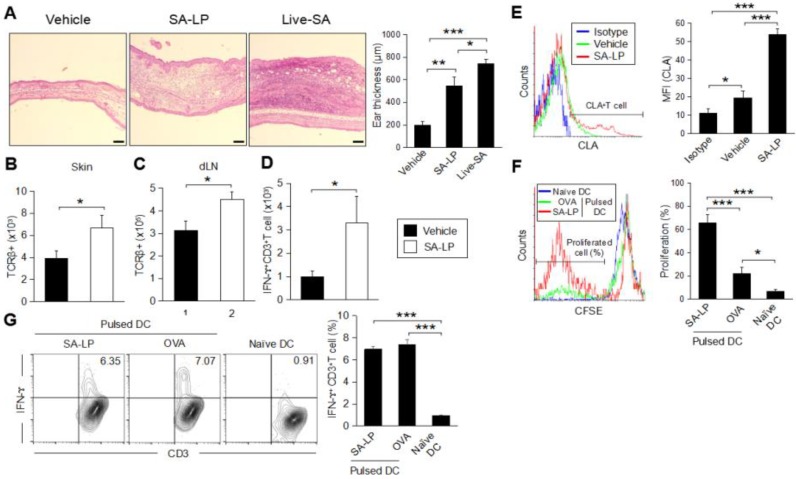
SA-LP induces skin inflammation, accompanied with antigen-specific effector T cell accumulation. (**A**–**E**) WT mice were treated with SA-LP (and live-SA) or vehicle solution (0.9% NaCl) by ID injection into the ear. After 120 hr of the treatment, the mice were used for each analysis. (**A**) Skin histology of the treated ear. Ear section was stained with H–E. The scale bar represents 100 μm. Thickness of the treated ear was measured from each section. (**B**,**C**) Total number of T cells on the treated skin (**B**) and skin-dLN (**C**). The TCRβ^+^ T cells were detected from skin-isolated leukocytes by flow cytometry. (**D**) Total number of IFN-ɤ-producing T cells on the SA-LP-treated ear. The isolated skin leukocytes were re-stimulated with PMA (100 ng/mL) and ionomycin (1 μg/mL). The cells were treated by intracellular staining for IFN-ɤ detection. The IFN-γ^+^CD3^+^T cells were detected from stained cells by flow cytometry. (**E**) CLA expression on the skin T cell in SA-LP mice. The isolated skin leukocytes were analyzed by flow cytometry. The CLA^+^ cells were detected from the population gated on CD3^+^T cells. The MFI of CLA was calculated from the histogram. (**F**) Ex vivo antigen re-stimulation for skin T cells. The skin leukocytes were isolated from SA-LP ID-injected ear, then the cells were labeled by CFSE. For re-stimulation, the cells were cocultured with antigen (SA-LP or LPS + OVA) activated or naive splenic DCs for 24 hr. The proliferated cells were detected from the population gated on CD3^+^ cells by flow cytometry. The T cell proliferation rate (%) was calculated from the histogram. (**G**) In vitro antigen presentation for naive T cells. Splenic DCs were stimulated with antigen (SA-LP or OVA + LPS) for 24 hr. The stimulated or naive DCs were cocultured with splenic naive T cells for 72 h. At the last 6 hr, the cells were re-stimulated with PMA (100 ng/mL) and ionomycin (1 μg/mL). The proliferated T cells were treated with intracellular staining for IFN-ɤ^+^CD3^+^T cell detection by flow cytometry. The percentage of IFN-ɤ-producing T cells was calculated from the flow cytometry image. (**B**–**F**) In each experiment, 3–5 mice were used for vehicle and SA-LP ID injection. The isolated cells from the ear and dLN were pooled, and used for flow cytometry analysis. Data are shown as the mean and SD of at least three samples of a single experiment, and are representative of at least three independent experiments. A Student’s *t*-test was used to analyze data for significant differences. Values of * *p* < 0.05, ** *p* < 0.01, and *** *p* < 0.001 were regarded as significant.

To investigate the effect of SA-LP-activated mDCs increasing in the SA-LP ID injected mice, we analyzed the phenotype related to the immune cell response in detail, especially focusing on T cells. Skin inflammation originating from *S. aureus* are usually characterized with abundant T cell accumulation [29]. The SA-LP ID injection established serious epidermal and dermal hyperplasia in the ear (Figure 3A). The ear thickness of SA-LP ID-injected mice was significantly severe to that of the vehicle control (Figure 3A). Even though it was less intense than the live-SA ID injected case, the tissue environment in the injected site was absolutely changed from a healthy condition. It means that a high rate of immune cell accumulation were observed in the SA-LP ID-injected site where T cell accumulation was especially significant (Figure 3B and Figure S4A). An increase in T cells was also observed in the dLN (Figure 3C). These accumulated T cells were suspected to work as IFN-γ-producing T cells, which has been generally observed in commensal bacteria-originated skin inflammation [2,30]. As per the evidence, the abundant IFN-γ-producing T cells were accumulated in the SA-LP-originated inflamed skin (Figure 3D). The skin accumulated-T cells strongly expressed cutaneous lymphocyte-associated antigen (CLA), which is a well-known regulator of T cell homing into the skin [31] (Figure 3E). These accumulated T cells were generated in an antigen-specific manner, which means that the skin-isolated T cells strongly proliferated by SA-LP in ex vivo antigen re-stimulation (Figure 3F). From these studies, we estimated that the accumulated T cells were derived from dLN through the priming effect by DCs for naive T cells in a SA-LP-specific manner. In vitro antigen presentation showed that SA-LP-activated DCs were successful in priming naive T cells, so that the IFN-γ-producing T cells were strongly induced in this system (Figure 3G). Thus, skin-accumulated T cells were derived from the priming effect of the SA-LP-activated DC, and work as IFN-ɤ-producing T cells to generate skin inflammation in the SA-LP ID-injected site.

### 2.4. SA-LP Induces IFN-ɤ-Producing CD4^+^ and CD8^+^T Cell Generation

**Figure 4:**
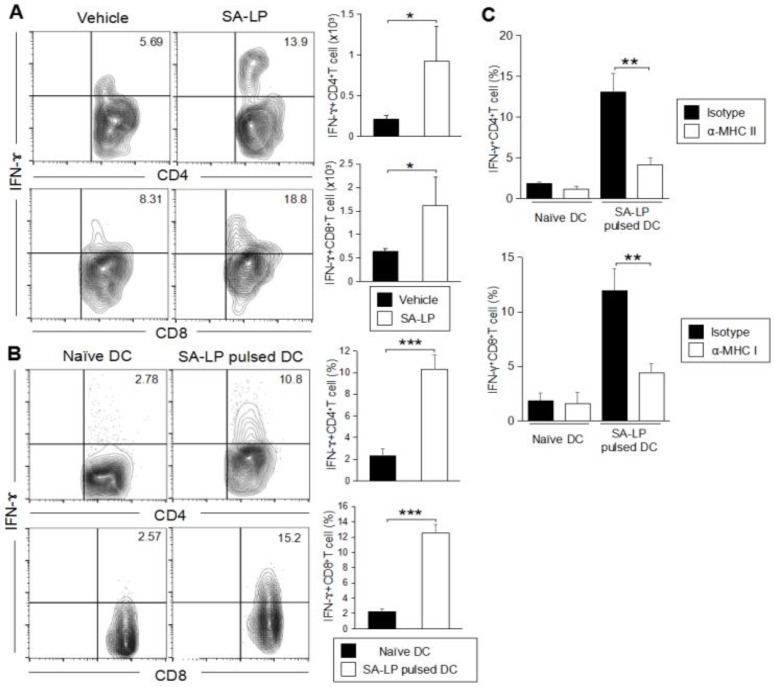
IFN-ɤ-producing T cells accumulate on the SA-LP ID injected skin. (**A**) Subset analysis for accumulated T cells in SA-LP ID-injected ear. WT mice were treated with SA-LP (10 μg) or vehicle solution (0.9% NaCl) by ID injection into the ear. After 120 hr of the treatment, the skin leukocytes were isolated from the ear, then the cells were re-stimulated with PMA (100 ng/mL) and ionomycin (1 μg/mL) for 6 hr. The stimulated cells were extracellularly and intracellularly stained for the detection of IFN-γ^+^ CD4^+^ and CD8^+^T cells in the population gated for CD3^+^ cells by flow cytometry. The total number of target T cells was calculated from the flow cytometry image. (**B**) In vitro antigen presentation for naive CD4^+^ and CD8^+^T cells. LN isolated DCs were stimulated with SA-LP (1 μg/mL) for 24 hr. The stimulated DCs were cocultured with splenic naive CD4^+^ or naive CD8^+^T cells for 72 hr. At the last 6 hr, the cells were re-stimulated with PMA (100 ng/mL) and ionomycin (1 μg/mL). The proliferated cells were extracellularly and intracellularly stained for the detection of IFN-γ^+^CD4^+^ and CD8^+^T cells in the population gated on CD3^+^ cells by flow cytometry. The total percentage of target cells was calculated from the flow cytometry image. (**C**) In vitro MHC blocking for DC. LN isolated DCs were pre-incubated with anti-MHC class I mAb (10 ug/mL) or anti-MHC class II mAb (10 ug/mL) for 1 hr. Then, the DCs were stimulated with SA-LP (1 μg/mL) for 24 hr in the presence of MHC blocking mAb. The stimulated DCs were used for coculture with splenic naive CD4^+^ or CD8^+^T cells for 72 h. At the last 6 hr, T cells were re-stimulated with PMA (100 ng/mL) and ionomycin (1 μg/mL). The T cells were extracellularly and intracellularly stained for the detection of IFN-γ^+^CD4^+^ and CD8^+^T cells in the population gated for CD3^+^ cells by flow cytometry. The total percentage target T cell was calculated from the flow cytometry image. (**A**) In each experiment, 3–5 mice were used for vehicle and SA-LP ID injection. The isolated cells from ear were pooled, and used for flow cytometric analysis. Data are shown as the mean and SD of at least three samples of a single experiment, and are representative of at least three independent experiments. A Student’s *t*-test was used to analyze data for significant differences. Values of * *p* < 0.05, ** *p* < 0.01, and *** *p* < 0.001 were regarded as significant.

After confirming the T cell accumulation on the inflamed skin in SA-LP ID-injected mice, our interest was directed to the subset of the T cells suspected to be responsible for the SA-LP-specific inflammatory response on the skin. In the SA-LP ID-injected mice, we performed characterization of the skin-accumulated T cell subset. The SA-LP ID injection upregulated not only CD4^+^T cells, but also CD8^+^T cells in the skin (Figure S4B). In order to deeply understand the increase of the specific Teff cell subset, we analyzed cytokine production in both the CD4^+^ and CD8^+^T cells. The SA-LP ID injection significantly induced IFN-ɤ-producing CD4^+^T cells (Th1) and CD8^+^T cells (Tc1) on the skin (Figure 4A and Figure S4C,D). The production of other cytokines such as IL-17A or IL-4 from the T cells were not determined as abundantly in the SA-LP ID-injected mice (Figure S4C,D). Other T cell subsets such as γδT, NKT, and Treg, did not show a significant increase in the SA-LP-treated skin (Figure S4E).

To reveal the detailed mechanism for the generation of Teff accumulated on the SA-LP ID injected skin, we investigated the antigen specificity for naive T cell priming. The coculture of SA-LP-activated DCs and splenic naive CD4^+^T cells showed the induction of IFN-γ producing CD4^+^T (Th1) cells (Figure 4B, upper). Similar to this event, SA-LP-activated DCs were successful in priming splenic naive CD8^+^T cells, and generated IFN-γ producing CD8^+^T (Tc1) cells (Figure 4B, lower). The skin-dLN in the SA-LP ID injected mice possessed a large number of Th1 and Tc1 cells when compared with the vehicle control (Figure S4F). This was strong proof that the skin-accumulated Teff originated from a priming effect dependent on SA-LP. Following these findings, we investigated further mechanisms of Teff generation by using SA-LP as an antigen. In a coculture of SA-LP-activated DCs and splenic naive CD4^+^T cells, MHC class II blocking mAb treatment inhibited Th1 generation (Figure 4C, upper). Similar to this result, MHC class I blocking mAb suppressed Tc1 generation (Figure 4C, lower). These results suggest that SA-LP is presented to naive CD4^+^ and CD8^+^T cells through DCs by antigen-presenting molecules, so that Th1 and Tc1 cells are generated by the priming effect, respectively. In particular, the Tc1 generation was regulated by a cross-presentation mechanism. Thus, the skin inflammation induced by SA-LP ID injection is attributed to Th1 and Tc1 accumulation on the skin.

### 2.5. DDC Plays an Important Role for the Induction of SA-LP-Originated Skin Inflammation

**Figure 5:**
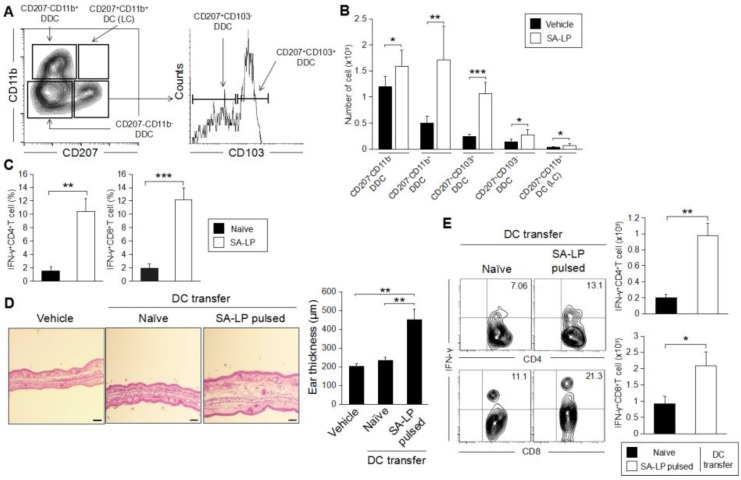
*SA*-LP-activated dermal dendritic cells have the responsibility of inducing skin inflammation. (**A**) The gating strategy of skin DC subset analysis. The mDC (MHC class II^hi^CD11c^+^) population was gated, then the population was separated to each subset by following CD207 and CD11b expression. The CD207^+^CD11b^−^ population was further separated with CD103 expression. Finally, skin DCs were classified as five subsets (CD207^−^CD11b^−^, CD207^−^CD11b^+^, CD207^+^CD103^+^, CD207^+^CD103^−^, and CD207^+^CD11b^+^ (Langerhans cell; LC)). (**B**) Activated DC subset classification on the SA-LP ID-injected mice. WT mice were treated with SA-LP (10 μg) or vehicle solution (0.9% NaCl) by ID injection into the ear. After 48 hr of treatment, the skin-dLN cells were isolated, and stained with anti-MHCII, anti-CD11c, anti-CD11b, anti-CD207, and anti-CD103 mAb. The stained cells were analyzed by flow cytometry by following the gating strategy (**A**). (**C**) Ex vivo antigen presentation for naive T cells. The DCs were isolated from skin-dLN in the SA-LP ID-injected mice. The DCs were cocultured with splenic naive T cells for 72 hr. For the last 6 hr, the proliferated cells were re-stimulated with PMA (100 ng/mL) and ionomycin (1 μg/mL). The cells were extracellularly and intracellularly stained for the detection of IFN-γ^+^CD4^+^, and CD8^+^T cells in the population gated for CD3^+^ cells by flow cytometry. (**D**,**E**) SA-LP-activated DC transfer induced skin inflammation bearing IFN-γ-producing T cells. The DCs were isolated from LN, and stimulated with SA-LP for 24 hr. The DCs were transferred into WT mice ears by ID injection. After 120 hr of treatment, the mice were used for analysis. (**D**) Skin histology of the treated ear. Ear section stained with H-E. The scale bar represents 100 μm. Thickness of the treated ear was measured from each section. (**E**) Subset analysis for accumulated T cells on the inflamed skin. Skin leukocytes were isolated from the ear, then the cells were re-stimulated with PMA (100 ng/mL) and ionomycin (1 μg/mL) for 6 hr. The stimulated cells were extracellularly and intracellularly stained for detection of IFN-γ^+^ CD4^+^ and CD8^+^T cells in the population gated for CD3^+^ cells by flow cytometry. The total number of target cells was calculated from the flow cytometry image. (**B**,**E**) In each experiment, 3–5 mice were used for vehicle and SA-LP ID injection. The isolated cells from the ear and skin-dLN were pooled, and used for flow cytometry analysis. Data are shown as the mean and SD of at least three samples of a single experiment, and are representative of at least three independent experiments. A Student’s *t*-test was used to analyze data for significant differences. Values of * *p* < 0.05, ** *p* < 0.01 and *** *p* < 0.001 were regarded as significant.

In the skin inflammatory response originating from pathogen infection, skin DCs are usually activated by a specific target [32]. The activated DCs migrate into skin-dLNs, and induce subsequent Teff generation through antigen presentation [33]. In this entire process, mDCs frequently show specificity in the subset driven by each substance, which is finally processed and is presented to naive T cells as an antigen [34]. Therefore, we characterized the mDC subset, which was specifically activated by SA-LP. Skin DCs are usually separated as five different subsets by focusing on the surface marker molecule such as CD207, CD11b and CD103 (Figure 5A) [35]. Following this approach, we analyzed each subset of mDC in the SA-LP ID-injected mice. We identified the dramatic increase of DDC (CD207^−^CD11b^−^, CD207^−^CD11b^+^, CD207^+^CD103^+^, and CD207^+^CD103^−^) in the mice (Figure 5B). To investigate the responsibility of SA-LP-activated mDCs for Teff generation, we performed an ex vivo antigen presentation assay (Figure S5). The DCs isolated from skin-dLN in the SA-LP ID injected mice strongly induced Th1 and Tc1 cells (Figure 5C). To investigate the importance of DC for the generation of SA-LP-originated skin inflammation, we performed an adoptive transfer of SA-LP-activated DCs by ID injection into the ear (Figure S6A). After 48 hr of the injection, the injected cells were detected in skin-dLN as mDCs (Figure S6B). After 120 hr of the treatment, the treated ear showed significant thickness when compared with the vehicle control and naive DC-injected mice (Figure 5D). The SA-LP-activated DC-transferred mice showed severe epidermal and dermal hyperplasia in the inflamed site. Furthermore, the treated skin was accompanied by abundant Th1 and Tc1 accumulation (Figure 5E). The DC-transferred mice also showed a significant increase of Th1 and Tc1 in the skin-dLN (Figure S6C), so that the skin-accumulated Teff was speculated as having been derived from the priming effect to naive T cells through the transferred SA-LP-activated DC. Taken together, DC activation derived from SA-LP is indispensable and triggers the initiation of skin inflammation established by Teff generation through the DCs, and their accumulation on the skin.

## 3. Discussion

The *S. aureus* cell wall is composed of several substances, and has been traditionally investigated as an antigen to induce the host immune response, and inflammation in each tissue and organ [36]. PGN and LTA are the most famous cell wall components in Gram-positive bacteria; therefore, many studies have focused on these substances and revealed the mechanisms behind induction of the inflammatory response [37,38]. According to these studies, we initially investigated the antigenicity of PGN and LTA in ID-injected mice. We found that PGN and LTA induced inflammation on the injected skin; however, there was much less severity of thickness when compared with whole bacteria (live or HK) injections (Figure 3A and Figure S3). In addition to the findings, PGN and LTA failed to induce the accumulation of abundant Teff on the treated skin, while granulocytes and macrophages were increased in a dominant manner (unpublished data). On the other hand, live-SA application showed severe epidermal and dermal hyperplasia caused by the accumulation of abundant Teff in the inflamed site (Figure 3A). This evidence provided the possibility of the existence of other dominant inflammation inducers in the *S. aureus* cell wall. Lipoprotein located on the *S. aureus* cell wall was revealed as a strong antigen from in vitro study by using BMDCs, and it generated serious inflammation on the treated skin (Figure 1 and Figure 3A). The inflammatory microenvironment in the SA-LP ID injected skin was similar to the live-SA treated case (Figure 3A), which means that SA-LP has a potentially important role in establishing systemic immune response in *S. aureus*-originated skin inflammation. Throughout the study, our results indicated the important role of DCs in SA-LP-derived skin inflammation. In the SA-LP ID-injected mice, a high population of mDC-loaded SA-LP as an antigen was observed in the skin-dLN (Figure 2C), and it possessed the responsibility of generating Teff accumulated on the inflamed site (Figure 3B–D and Figure S4A). Ex vivo antigen presentation also showed the antigenicity of SA-LP in DCs for the generation of Th1 and Tc1 (Figure 5C). Hence, we concluded that SA-LP is a strong factor in establishing skin inflammation, rather than other cell wall components, in *S. aureus*.

Although we confirmed that SA-LP induces severe skin inflammation, we also considered its antigenicity for other substances derived from *S. aureus*, as SA-LP failed to induce an increase in Th17 or γδT, which is generally observed in *S. aureus*-related skin inflammation [7,8]. These T cell subsets were slightly increased in the SA-LP ID injected mice, but there was not a dramatic change when compared with the vehicle control (Figure S4C,E). Notably, we found that LTA promoted γδT and NKT cell accumulation on the ID injected skin (unpublished data). The exact mechanism for the increase of these T cell subsets are still unknown; however, it is obvious that several factors are orchestrated to establish a whole T cell-mediated response in *S. aureus*-related skin inflammation. Furthermore, several cell wall components and toxins in *S. aureus* also induced skin inflammation with accumulation and activation of various cells [39,40,41]. These responses also seem to be important factors for generating total inflammatory responses against for *S. aureus*. We have suspected that it is the reason for why SA-LP ID injection showed less severity for skin inflammation compared with cases with live *S. aureus* applied.

When we detected a strong induction of Th1 and Tc1 on the skin, and dLN in the SA-LP ID-injected mice (Figure 4A and Figure S4C,D), we suspected the existence of a relationship with memory T cells (T_CM_, T_E__M_ and T_RM_) as previous reports have indicated that commensal bacteria possesses the ability to establish a memory T cell pool in LN and peripheral organs, including the skin [2,42]. In fact, both Th1 and Tc1 were detected in the vehicle control mice, though these absolute numbers were less than that of the SA-LP treated mice (Figure 4A and Figure S4C,D,F). The skin-accumulated T cells did not express CD69 and CD103, common T_RM_ markers, in the SA-LP ID injected mice. Furthermore, the T cells detected in dLN did not contain the CD62L^+^CCR7^+^ (T_CM_) population (unpublished data). Taken together, we concluded that the accumulated Teff on the SA-LP ID-injected skin mainly originated from the priming effect for naive T cells in the skin-dLN through the SA-LP-activated DCs.

In this study, we revealed that DDCs have the responsibility of generating Teff through specific recognition of SA-LP. It has already been reported that each DDC has a special character for Teff generation [43,44,45]. As a general priming mechanism for CD4^+^ naive T cells, these DDCs have strong activity. On the other hand, a specific DDC subset such as CD207^+^CD103^+^DDC was revealed as being important for cross-presentation, which is an indispensable mechanism for the generation of CD8^+^Teff [45,46]. Our finding is strongly supported by these reports, as the SA-LP ID injected mice showed a significant increase in mDDCs in the skin-dLN, and the DCs strongly induced the generation of Th1 and Tc1 with high specificity (Figure 4A and Figure S4C,D,F). Furthermore, the evidence of antigen presentation through both MHC class II for naive CD4^+^T cells and MHC class I for naive CD8^+^T cells were also confirmed by MHC blocking for in vitro antigen presentation using SA-LP as an antigen (Figure 4C). However, we have not yet revealed the exact mechanism of the cross-presentation for SA-LP by CD207^+^CD103^+^DDC. To reveal this mechanism, further investigation is required.

Throughout this study, we have revealed that the DDC and Teff axis is a major immunological response to establish skin inflammation originating from the *S. aureus* lipoprotein. Our findings imply that lipoprotein produced from Gram-positive bacteria, including *S. aureus*, is one of the strong potential inflammation inducers on the skin. This study is the first comprehensive report for the relationship of bacterial lipoprotein and skin inflammation. Furthermore, this study used an in vivo approach to reveal the mechanism of total immune response against bacterial lipoprotein, so it has a high impact for the novel understanding of *S. aureus* pathogenesis on the skin. Our data suggested that *S. aureus* lipoprotein may be a therapeutic target, so that it has a chance to be a novel approach for the treatment of serious acute/chronicle skin inflammation originating with *S. aureus*. The immunological character of *S. aureus* lipoprotein still has several unclear parts; however, our findings will be a great help for the strategy of prevention and cure in *S. aureus*-related serious skin inflammation.

## 4. Materials and Methods

Mice. C57BL/6J specific pathogen free (SPF) mice were purchased from The Jackson Laboratory (Bar Harbor, ME, USA). Gender matched mice between 8–12 weeks of age were used for each experiment.

Reagents and antibodies. Lipoteichoic acid (LTA, *S. aureus* origin), peptidoglycan (PGN, *S. aureus* origin), Pam3CSK4, sparstolonin B (SsnB), phorbol 12-myristate 13-acetate (PMA), ionomycin, ovalbumin (OVA) and α-galactosylceramide (α-GalCer) were all purchased from Sigma Aldrich (St Louis, MO, USA). Dispase and collagenase were purchased from Thermo Fisher Scientific (Waltham, MA, USA). Percoll was purchased from GE Healthcare (Chicago, IL, US). The Cytofix/Cytoperm kit was purchased from BD Bioscience (Franklin Lakes, NJ, USA). Recombinant murine granulocyte macrophage-colony stimulating factor (rmGM-CSF) was purchased from Peprotech (Rocky Hill, NJ, USA). Anti-CD11c (N418), anti-CD11b (M1/70), anti-CD207 (4C7), anti-CD103 (2E7), anti-CCR7 (4B12), anti-CLA (HECA-452), anti-CD80 (16-010A1), anti-CD86 (GL-1), anti-CD45 (30-F11), anti-CD3 (17A2), anti-CD4 (GK1.5), anti-IL-17A (BL168), anti-CD16/CD32 (2.4G2) (93), and 5-(and -6)-carboxyfluorescein diacetate succinimidyl ester (CFSE) were all purchased from Biolegend (San Diego, CA, USA). Anti-MHC-II (M5/114.15.2), anti-CD11b (M1/70), anti-TCRβ (H57–597), anti-ɤδTCR (GL3), anti-IFN-γ (XMG1.2), anti-IL-4 (8D4–8), and anti-Toll-like receptor 2 (TLR2) were all purchased from Thermo Fisher Scientific (Waltham, MA, USA). CD1d-tetramer was purchased from Proimmune (Oxford, UK). Anti-MHC class I (HB159) and anti-MHC class II (M5/114) was purchased from Bio X Cell (West Lebanon, NH, USA). The isotype-matched control for each antibody was purchased from the same company.

*S. aureus* culture. The frozen *S. aureus* (MRSA; USA300) stock was thawed on ice, then transferred to a tryptic soy broth (TSB; BD bioscience, Franklin Lakes, NJ, USA) and cultured at 37 °C for 18 hr with shaking. The colony forming units (CFU) were calculated in each culture. Heat-killed *S. aureus* (HK-SA) was prepared with heating at 95 °C for 30 min. The heated *S. aureus* suspension was centrifuged at 10,000 rpm for 1 min to harvest the bacteria cells, then the cell pellet was resuspended in phosphate buffered saline (PBS) or 0.9% NaCl.

Lipoprotein isolation and preparation of the cell wall component from *S. aureus.* Lipoprotein was isolated from *S. aureus* by following a method described in previous reports with modifications [18,19,23]. Briefly, cultured *S. aureus* (10^7–8^ CFU/mL) was harvested by centrifuging at 5000× *g* for 20 min. The pellet was washed twice by 20 mM Tris-HCl (pH 8.0). The pellet was resuspended in 20 mM Tris–HCl (pH 8.0), then the bacterial cell was crushed with 0.3 mm stainless beads. The treated suspension was centrifuged at 5000× *g* for 20 min, then the supernatant was harvested as the protein suspension. The suspension was mixed with 100% ethanol and kept at −20 °C overnight. The sample was centrifuged at 12,000× *g* for 15 min, then the precipitated pellet was washed with 80% ethanol and centrifuged again at 12,000× *g* for 5 min. The precipitated pellet was dissolved with 1 M urea/50 mM Tris–HCl, 50 mM ethylenediaminetetraacetic acid (EDTA) (pH 8.0) (Crude Protein Extract; CPE). Triton X-114 was added to the protein suspension (final 1%), then the suspension was incubated at 4 °C with gentle mixing. The incubated suspension was heated at 37 °C, forming the micelle phase-containing lipoprotein. The micelle phase was extracted and lipoprotein (Clude *S. aureus*-lipoprotein; SA-LP) was harvested by following a method for CPE precipitation. The SA-LP was separated to each fraction (L1 to L4) in a size dependent manner by using a molecular weight cut-off filter (Amicon ultra; Darmstadt, Germany). For preparation of the cell wall extract (CWE), the twice-washed *S. aureus* pellet was resuspended in 20 mM Tris–HCl (pH 8.0). The suspension was kept at −80 °C for 30 min, then sonicated for 20 min. The suspension was centrifuged at 5000× *g* for 20 min, and the pellet was harvested as CWE.

Sodium dodecyl sulfate-poly-acrylamide gel electrophoresis (SDS-PAGE) and silver stain. The extracted protein solution was diluted with 5 × SDS sample buffer (2% SDS, 62.5 mM Tris–HCl (pH 6.8), 10% glycerol, 0.01% bromophenol blue, 50 mM dithiothreitol (DTT). The proteins, separated by SDS-PAGE, were visualized with a Silver Stain Kit (Thermo Fisher Scientific, Waltham, MA, USA). Whole staining procedure was followed with the manual.

Mouse primary cell isolation. Skin leukocytes were isolated by following a method described in a previous report with modification [47]. Briefly, the extracted ear was washed with tissue washing buffer (RPMI 1640 supplemented with 10% fetal bovine serum (FBS), 10 mM 4-(2-hydroxyethyl)-1-piperazineethanesulfonic acid (HEPES) and 100 U/mL penicillin, 100 mg/mL streptomycin) at 37 °C for 30 min with gentle shaking. The ear was separated into the ventral and dorsal sheets from the cartilage, and incubated at 4 °C overnight with dispase working solution (tissue washing buffer containing 0.25 mg/mL of dispase) to separate the epidermal and dermal sheets. These sheets were chopped with scissors, then incubated at 37 °C for 30 min in collagenase working solution (tissue washing buffer containing 1 mg/mL collagenase and 0.01% DNase). The digested ear pieces were passed through a 5 mL syringe with a 22 G needle to make single cell suspensions. Lymph node cells were prepared from skin-draining LN (dLN) by following a method described in a previous report [48]. Briefly, isolated dLN was crushed on a dish and suspended in cell culture medium. The cell suspension was filtered through a 70 μm cell strainer, then twice washed with cell culture medium (RPMI 1640 supplemented with 10% fetal bovine serum (FBS), 100 U/mL penicillin and 100 mg/mL streptomycin). Splenocytes were obtained from the spleen by following a method described in a previous report [48]. Briefly, isolated spleen was crushed on a 70 μm cell strainer, and the cells were suspended in cell culture medium. After being washed once with the cell culture medium, the cells were further resuspended in an erythrocyte lysis solution (155 mM NH_4_Cl, 10 mM KHCO_3_, 1 mM Na-EDTA, and 17 mM Tris–HCl (pH 7.3)). After being washed twice with cell culture medium, the cells were used as splenocytes. Mouse bone marrow leukocytes were obtained from the tibia and femur. After extracting the tibia and femur, bone marrow leukocytes were flushed out with a syringe containing cell culture medium. The cell suspension was filtered through a 70 μm cell strainer and washed once with cell culture medium, then the cells were treated with the erythrocytes lysis solution. After lysis, the cells were washed twice with cell culture medium, and the cells were used as bone marrow leukocytes. Pan-naïve T cells were isolated from the splenocyte by using an EasySep Mouse Pan-Naïve T Cell Isolation Kit (Stemcell Technology; Vancouver, BC, Canada). Naïve CD4^+^ and CD8^+^T cells were isolated from the splenocyte by using a MagniSort mouse CD4 naïve T cell or mouse CD8 naïve T cell Enrichment kit (Thermo Fisher Scientific, Waltham, MA, USA), respectively. LN and splenic dendritic cells were isolated by using MagniSort Mouse CD11c Positive Selection kit (Thermo Fisher Scientific, Waltham, MA, USA). The whole procedure for the cell isolation kit was performed by following the manual.

Mouse BMDCs preparation. Mouse BMDCs were prepared by following a method described in a previous report [49]. At day 0, 2.0 × 10^6^ of bone marrow leukocytes were suspended in 10 mL of DC culture medium (cell culture medium containing 20 ng/mL of rmGM-CSF), and the cells were seeded on a 100 mm dish. At day 3, 10 mL of the fresh DC culture medium was added to the cultured cells. At days 6 and 8, half of the cultured medium was collected and centrifuged, then the cell pellets were resuspended in 10 mL of the fresh DC culture medium. The cell suspension was put back into the original plate. At day 10, cells were ready to use for each experiment.

BMDC stimulation assay. Naïve BMDCs were seeded on a 6-well plate with DC culture medium, then the cells were stimulated by the *S. aureus*-derived component, LTA (10 μg/mL), PGN (10 μg/mL), SA-LP (10 μg/mL or identical amounts with isolated from 10^6^ of *S. aureus*), CWE (identical amount with isolated from 10^6^ of *S. aureus*), CPE (10 μg/m or identical amount with isolated from 10^6^ of *S. aureus*) and live-SA (10^6^) and HK-SA (10^6^) for 24 hr. The TLR2 agonist Pam3CSK4 (500 ng/mL) and TLR4 agonist LPS (100 ng/mL) were used for the positive control. TLR2 was blocked by the anti-mouse TLR2 monoclonal antibody (mAB) (10 μg/mL) or inhibited by Ssn B (100 μM). The stimulated cells were analyzed by flow cytometry for the detection of activation markers (CD80, DC86 and MHC class II). Cultured medium was harvested for the measurement of cytokine production by ELISA.

Flow cytometry. Cell surface markers and intracellular cytokines were analyzed by a flow cytometer (FACScalibur and LSR-II; BD Biosciences, Franklin Lakes, NJ, USA) with the fluorochrome-conjugated monoclonal antibodies described in reagents and antibodies. The cells were initially incubated with FcR blocker (anti-CD16/32; 2.4G2) at 4 °C for 10 min. For surface marker staining, the cells were incubated with the antibody at 4 °C for 30 min. Intracelluler cytokine staining was performed by using a Cytofix/CytoPerm Kit (BD Biosciences, Franklin Lakes, NJ, USA) by following the manual. Briefly, the cells stained with the antibody for the surface marker were fixed and permilized. The cells were incubated with the antibody for cytokine staining at 4 °C for 30 min. The dead cells were excluded by forward scatter, side scatter, and propidium iodide gating. All data were analyzed by BD FACS Diva (BD bioscience, Franklin Lakes, NJ, USA) or FlowJo (Tree Star; Ashland, OR, USA).

Murine skin inflammation model. To establish the skin inflammation model, anesthetized mice were treated with the antigen by intradermal (ID) injection into the ear (Figure S2). The antigen was dissolved or diluted with 0.9% saline, then applied into the dorsal side of the ear. After 48 or 120 hr of the treatment, the mice were sacrificed and the treated ear and skin-dLN was excised for use in each analysis.

In vivo antigen tracking. For antigen tracking, FITC-labeled SA-LP and OVA or non-labeled OVA (10 μg in each) were ID-injected into the mouse ear. After 48 hr of the treatment, the skin dLN was extracted from the treated mice, then the isolated LN cells were analyzed by flow cytometry.

Ex vivo antigen re-stimulation. The skin leukocytes were isolated from the SA-LP ID-injected mice ears, then the cells were labeled by CFSE. The labeled cells were cocultured with stimulated splenic DCs (SA-LP 1 μg/mL or LPS 100 ng/mL + OVA 10 μg/mL) or naïve splenic DCs at 37 °C for 24 hr. The proliferated cells were analyzed by flow cytometry.

In vitro antigen presentation. Isolated primary DCs were stimulated with SA-LP (10 μg/mL) overnight. The stimulated or naive DCs were cocultured with pan-naïve T, naïve CD4^+^, or CD8^+^T cells for 72 hr. At the last 6 hr of the coculture, the proliferated cells were re-stimulated with 100 ng/mL of PMA, 1 μg/mL of ionomycin, and protein transportation was inhibited with Golgi stop (BD Bioscience). The proliferated cells were analyzed by flow cytometry.

In vitro MHC blocking assay. MHC class I and II molecules on the DCs were blocked with antibodies by following a method described in a previous report with modification [50]. Briefly, LN isolated DCs were pre-incubated with the blocking antibody for MHC class I or MHC class II (10 μg/mL in each) for 1 hr. An isotype antibody was also used for the control. Then, the cells were stimulated with SA-LP (10 μg/mL) overnight. The stimulated DCs were cocultured with splenic naïve CD4^+^ or CD8^+^T cells for 72 hr in the presence of the blocking antibody or isotype antibody. At the last 6 hr of the coculture, the proliferated cells were re-stimulated with 100 ng/mL of PMA, 1 μg/mL of ionomycin, and protein transportation was inhibited with Golgi stop (BD Bioscience). The cells were analyzed by flow cytometry.

SA-LP-activated dendritic cell transfer. The primary DCs were isolated from WT mouse LN, then the cells were labeled with CFSE and stimulated with SA-LP (10 μg/mL) at 37 °C overnight. The treated DCs were washed with cell culture medium three times, then the cells (2.0 × 10^6^) were transferred into WT mice ears by ID injection. After 48 and 120 hr of the treatment, the skin-dLN and ear were used for each analysis.

Cytokine measurement by Enzyme-Linked Immuno Sorbent Assay (ELISA)*.* The cytokine (TNF-α, IL-12p40 and IL-6) produced from the stimulated cell was measured by using a Mouse ELISA kit (Thermo Fisher Scientific, Waltham, MA, USA) for each target. The whole procedure was performed by following the manual.

Statistical analyses. A Student’s *t*-test was used to analyze the data for significant differences. Values of * *p* < 0.05, ** *p* < 0.01, and *** *p* < 0.001 were regarded as significant.

## Supplementary Materials

The following are available online at http://www.mdpi.com/2076-0817/7/3/64/s1. Figure S1: Gel image of extracted *S. aureus* lipoprotein and antigenicity of size-separated SA-LP fraction in BMDCs. Figure S2: Murine skin inflammation model. Figure S3: *S. aureus* cell wall component, LTA and PGN, induce absolute skin inflammation, however these are significantly less than SA-LP case. Figure S4: Immune cell characterization in the SA-LP ID injected mice. Figure S5: In vitro antigen presentation for naive T cell. Figure S6: SA-LP activated DC transfer induces inflammation on the skin.

## References

[1] Cogen A.L., Nizet V., Gallo R.L. (2008). Skin microbiota: A source of disease or defence?. Br. J. Dermatol..

[2] Naik S., Bouladoux N., Linehan J.L., Han S.J., Harrison O.J., Wilhelm C., Conlan S., Himmelfarb S., Byrd A.L., Deming C. (2015). Commensal-dendritic-cell interaction specifies a unique protective skin immune signature. Nature.

[3] Kennedy E.A., Connolly J., Hourihane J.O., Fallon P.G., McLean W.H.I., Murray D., Jo J.H., Segre J.A., Kong H.H., Irvine A.D. (2017). Skin microbiome before development of atopic dermatitis: Early colonization with commensal *staphylococci* at 2 months is associated with a lower risk of atopic dermatitis at 1 year. J. Allergy Clin. Immunol..

[4] Nakatsuji T., Chen T.H., Narala S., Chun K.A., Two A.M., Yun T., Shafiq F., Kotol P.F., Bouslimani A., Melnik A.V. (2017). Antimicrobials from human skin commensal bacteria protect against *Staphylococcus aureus* and are deficient in atopic dermatitis. Sci. Transl. Med..

[5] Biedermann T., Skabytska Y., Kaesler S., Volz T. (2015). Regulation of T Cell Immunity in Atopic Dermatitis by Microbes: The Yin and Yang of Cutaneous Inflammation. Front. Immunol..

[6] Liu H., Archer N.K., Dillen C.A., Wang Y., Ashbaugh A.G., Ortines R.V., Kao T., Lee S.K., Cai S.S., Miller R.J. (2017). *Staphylococcus aureus* Epicutaneous Exposure Drives Skin Inflammation via IL-36-Mediated T Cell Responses. Cell Host Microbe.

[7] Laborel-Préneron E., Bianchi P., Boralevi F., Lehours P., Fraysse F., Morice-Picard F., Sugai M., Sato’o Y., Badiou C., Lina G. (2015). Effects of the *Staphylococcus aureus* and *Staphylococcus epidermidis* Secretomes Isolated from the Skin Microbiota of Atopic Children on CD4^+^ T Cell Activation. PLoS ONE.

[8] Mazmanian S.K., Liu C.H., Tzianabos A.O., Kasper D.L. (2005). An immunomodulatory molecule of symbiotic bacteria directs maturation of the host immune system. Cell.

[9] Boguniewicz M., Leung D.Y. (2011). Atopic dermatitis: A disease of altered skin barrier and immune dysregulation. Immunol. Rev..

[10] De Benedetto A., Kubo A., Beck L.A. (2012). Skin barrier disruption: A requirement for allergen sensitization?. J. Investig. Dermatol..

[11] Atilano M.L., Pereira P.M., Yates J., Reed P., Veiga H., Pinho M.G., Filipe S.R. (2010). Teichoic acids are temporal and spatial regulators of peptidoglycan cross-linking in *Staphylococcus aureus*. Proc. Natl. Acad. Sci. USA.

[12] Qamar A., Golemi-Kotra D. (2012). Dual roles of FmtA in *Staphylococcus aureus* cell wall biosynthesis and autolysis. Antimicrob. Agents Chemother..

[13] Sobhanifar S., Worrall L.J., Gruninger R.J., Wasney G.A., Blaukopf M., Baumann L., Lameignere E., Solomonson M., Brown E.D., Withers S.G. (2015). Structure and mechanism of *Staphylococcus aureus* TarM, the wall teichoic acid α-glycosyltransferase. Proc. Natl. Acad. Sci. USA.

[14] Bubeck Wardenburg J., Williams W.A., Missiakas D. (2006). Host defenses against *Staphylococcus aureus* infection require recognition of bacterial lipoproteins. Proc. Natl. Acad. Sci. USA.

[15] Shahmirzadi S.V., Nguyen M.T., Götz F. (2016). Evaluation of *Staphylococcus aureus* Lipoproteins: Role in Nutritional Acquisition and Pathogenicity. Front. Microbiol..

[16] Dziarski R., Gupta D. (2005). *Staphylococcus aureus* peptidoglycan is a toll-like receptor 2 activator: A reevaluation. Infect. Immun..

[17] Schwandner R., Dziarski R., Wesche H., Rothe M., Kirschning C.J. (1999). Peptidoglycan- and lipoteichoic acid-induced cell activation is mediated by toll-like receptor 2. J. Biol. Chem..

[18] Tawaratsumida K., Furuyashiki M., Katsumoto M., Fujimoto Y., Fukase K., Suda Y., Hashimoto M. (2009). Characterization of N-terminal structure of TLR2-activating lipoprotein in *Staphylococcus aureus*. J. Biol. Chem..

[19] Hashimoto M., Tawaratsumida K., Kariya H., Aoyama K., Tamura T., Suda Y. (2006). Lipoprotein is a predominant Toll-like receptor 2 ligand in *Staphylococcus aureus* cell wall components. Int. Immunol..

[20] Schmaler M., Jann N.J., Ferracin F., Landolt L.Z., Biswas L., Götz F., Landmann R. (2009). Lipoproteins in *Staphylococcus aureus* mediate inflammation by TLR2 and iron-dependent growth in vivo. J. Immunol..

[21] Zaba L.C., Krueger J.G., Lowes M.A. (2009). Resident and “inflammatory” dendritic cells in human skin. J. Investig. Dermatol..

[22] Akira S., Uematsu S., Takeuchi O. (2006). Pathogen Recognition and Innate Immunity. Cell.

[23] Kapsenberg M.L. (2003). Dendritic-cell control of pathogen-driven T-cell polarization. Nat. Rev. Immunol..

[24] Stoll H., Dengjel J., Nerz C., Götz F. (2005). *Staphylococcus aureus* deficient in lipidation of prelipoproteins is attenuated in growth and immune activation. Infect. Immun..

[25] Fournier B. (2013). The function of TLR2 during staphylococcal diseases. Front. Cell. Infect. Microbiol..

[26] Liang Q., Wu Q., Jiang J., Duan J., Wang C., Smith M.D., Lu H., Wang Q., Nagarkatti P., Fan D. (2011). Characterization of sparstolonin B, a Chinese herb-derived compound, as a selective Toll-like receptor antagonist with potent anti-inflammatory properties. J. Biol. Chem..

[27] Clatworthy M.R., Aronin C.E., Mathews R.J., Morgan N.Y., Smith K.G., Germain R.N. (2014). Immune complexes stimulate CCR7-dependent dendritic cell migration to lymph nodes. Nat. Med..

[28] Acton S.E., Astarita J.L., Malhotra D., Lukacs-Kornek V., Franz B., Hess P.R., Jakus Z., Kuligowski M., Fletcher A.L., Elpek K.G. (2012). Podoplanin-rich stromal networks induce dendritic cell motility via activation of the C-type lectin receptor CLEC-2. Immunity.

[29] Bröker B.M., Mrochen D., Péton V. (2016). The T Cell Response to *Staphylococcus aureus*. Pathogens.

[30] Shen W., Li W., Hixon J.A., Bouladoux N., Belkaid Y., Dzutzev A., Durum S.K. (2014). Adaptive immunity to murine skin commensals. Proc. Natl. Acad. Sci. USA.

[31] Leung D.Y., Gately M., Trumble A., Ferguson-Darnell B., Schlievert P.M., Picker L.J. (1995). Bacterial superantigens induce T cell expression of the skin-selective homing receptor, the cutaneous lymphocyte-associated antigen, via stimulation of interleukin 12 production. J. Exp. Med..

[32] Chong S.Z., Evrard M., Ng L.G. (2013). Lights, camera, and action: Vertebrate skin sets the stage for immune cell interaction with arthropod-vectored pathogens. Front. Immunol..

[33] Münz C., Steinman R.M., Fujii S. (2005). Dendritic cell maturation by innate lymphocytes: Coordinated stimulation of innate and adaptive immunity. J. Exp. Med..

[34] Clausen B.E., Stoitzner P. (2015). Functional Specialization of Skin Dendritic Cell Subsets in Regulating T Cell Responses. Front. Immunol..

[35] Henri S., Poulin L.F., Tamoutounour S., Ardouin L., Guilliams M., de Bovis B., Devilard E., Viret C., Azukizawa H., Kissenpfennig A. (2010). CD207^+^ CD103^+^ dermal dendritic cells cross-present keratinocyte-derived antigens irrespective of the presence of Langerhans cells. J. Exp. Med..

[36] Yoshimura A., Lien E., Ingalls R.R., Tuomanen E., Dziarski R., Golenbock D. (1999). Cutting edge: Recognition of Gram-positive bacterial cell wall components by the innate immune system occurs via Toll-like receptor 2. J. Immunol..

[37] Li C., Li H., Jiang Z., Zhang T., Wang Y., Li Z., Wu Y., Ji S., Xiao S., Ryffel B. (2014). Interleukin-33 increases antibacterial defense by activation of inducible nitric oxide synthase in skin. PLoS Pathog..

[38] Weidenmaier C., Peschel A. (2008). Teichoic acids and related cell-wall glycopolymers in Gram-positive physiology and host interactions. Nat. Rev. Microbiol..

[39] Lacey K.A., Geoghegan J.A., McLoughlin R.M. (2016). The Role of *Staphylococcus aureus* Virulence Factors in Skin Infection and Their Potential as Vaccine Antigens. Pathogens.

[40] Nakamura Y., Oscherwitz J., Cease K.B., Chan S.M., Muñoz-Planillo R., Hasegawa M., Villaruz A.E., Cheung G.Y., McGavin M.J., Travers J.B. (2013). *Staphylococcus* δ-toxin induces allergic skin disease by activating mast cells. Nature.

[41] Hepburn L., Hijnen D.J., Sellman B.R., Mustelin T., Sleeman M.A., May R.D., Strickland I. (2017). The complex biology and contribution of Staphylococcus aureus in atopic dermatitis, current and future therapies. Br. J. Dermatol..

[42] Nemoto Y., Kanai T., Kameyama K., Shinohara T., Sakamoto N., Totsuka T., Okamoto R., Tsuchiya K., Nakamura T., Sudo T. (2009). Long-lived colitogenic CD4^+^ memory T cells residing outside the intestine participate in the perpetuation of chronic colitis. J. Immunol..

[43] Haniffa M., Gunawan M., Jardine L. (2015). Human skin dendritic cells in health and disease. J. Dermatol. Sci..

[44] Sen D., Forrest L., Kepler T.B., Parker I., Cahalan M.D. (2010). Selective and site-specific mobilization of dermal dendritic cells and Langerhans cells by Th1- and Th2-polarizing adjuvants. Proc. Natl. Acad. Sci. USA.

[45] Nizza S.T., Campbell J.J. (2014). CD11b^+^ migratory dendritic cells mediate CD8 T cell cross-priming and cutaneous imprinting after topical immunization. PLoS ONE.

[46] Bedoui S., Whitney P.G., Waithman J., Eidsmo L., Wakim L., Caminschi I., Allan R.S., Wojtasiak M., Shortman K., Carbone F.R. (2009). Cross-presentation of viral and self antigens by skin-derived CD103^+^ dendritic cells. Nat. Immunol..

[47] Singh T.P., Zhang H.H., Borek I., Wolf P., Hedrick M.N., Singh S.P., Kelsall B.L., Clausen B.E., Farber J.M. (2016). Monocyte-derived inflammatory Langerhans cells and dermal dendritic cells mediate psoriasis-like inflammation. Nat. Commun..

[48] Saito S., Kawamura T., Higuchi M., Kobayashi T., Yoshita-Takahashi M., Yamazaki M., Abe M., Sakimura K., Kanda Y., Kawamura H. (2015). RASAL3, a novel hematopoietic RasGAP protein, regulates the number and functions of NKT cells. Eur. J. Immunol..

[49] Lutz M.B., Kukutsch N., Ogilvie A.L., Rössner S., Koch F., Romani N., Schuler G. (1999). An advanced culture method for generating large quantities of highly pure dendritic cells from mouse bone marrow. J. Immunol. Methods.

[50] Suurmond J., van Heemst J., van Heiningen J., Dorjée A.L., Schilham M.W., van der Beek F.B., Huizinga T.W., Schuerwegh A.J., Toes R.E. (2013). Communication between human mast cells and CD4^+^T cells through antigen-dependent interactions. Eur. J. Immunol..

