# Supplementary material for: Staphylococcus aureus Lipoprotein Induces Skin Inflammation, Accompanied with IFN-γ-Producing T Cell Accumulation through Dermal Dendritic Cells
Source: Pathogens. 2018 Jul 29;7(3):64. doi: 10.3390/pathogens7030064 (PMC6161079; doi:10.3390/pathogens7030064)
Supplement: Supplementary file 1 [file pathogens-07-00064-s001.pdf]

pathogens

MDPI

Supplementary Materials: The following are available online at www.mdpi.com/xxx/s1, Figure S1: title, Table S1: title, Video S1: title.

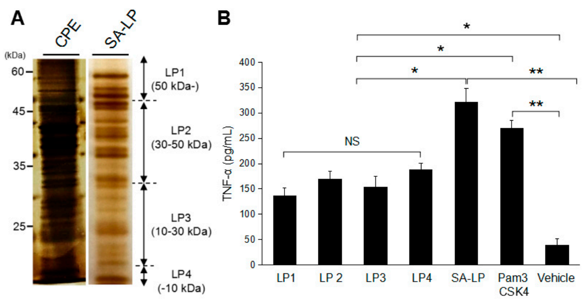

Figure S1. Gel image of extracted *S. aureus* lipoprotein and antigenicity of size-separated SA-LP fraction in BMDCs. (A) The CPE and SA-LP (from 10^8 of *S. aureus*) was separated by SDS-PAGE, and the gel was treated with silver stain. (B) SA-LP was fractionated by molecular weight cut-off filter by size dependent manner (LP1-4). BMDCs were stimulated with these LP (1 μg/mL), SA-LP (1 μg/mL) or Pam3CSK4 (500 ng/mL) for 24 h. The cytokine production in the cultured medium was measured by ELISA. Data are shown as the SD of at least three samples of a single experiment and are representative of at least three independent experiments. The Student's *t*-test was used to analyze data for significant differences. Values of *$p < 0.05$, **$p < 0.01$ and ***$p < 0.001$ were regarded as significant. NS; not-significant.

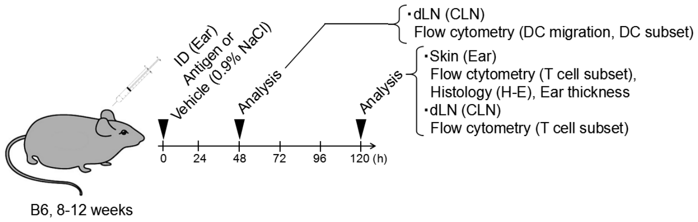

Figure S2. Murine skin inflammation model. WT mice (C57B6L/6J background) were treated with antigen or vehicle solution (0.9% NaCl) by ID injection into the ear. After 48 or 120 h of the treatment, the mice were sacrificed, then used for each analysis. The treated ear pieces were used for histological analysis, and the isolated skin and dLN cells were used for flow cytometry.

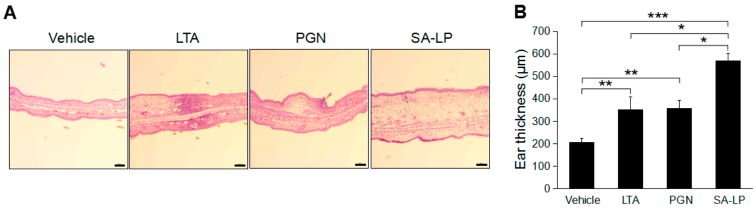

Figure S3. *S. aureus* cell wall component, LTA and PGN, induce absolute skin inflammation, however these are significantly less than SA-LP case. WT mice were treated with LTA (20 μg), PGN (20 μg), SA-LP (10 μg) or vehicle solution (0.9% NaCl) by ID injection into the ear. After 120 h of the treatment, the mice was used for analysis. (A) Skin histology of the treated ear. Ear section was stained with H-E. The scale bar represents 100 μm. (B) Thickness of the treated ear. Data are shown as the SD of at least three samples of a single experiment and are representative of at least three independent experiments. The Student's *t*-test was used to analyze data for significant differences. Values of *$p < 0.05$, **$p < 0.01$ and ***$p < 0.001$ were regarded as significant.

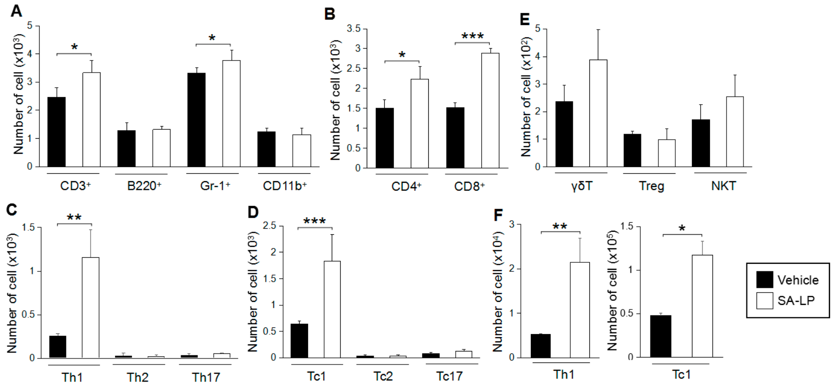

Figure S4. Immune cell characterization in the SA-LP ID injected mice. WT mice were treated with SA-LP (10 μg) or vehicle solution (0.9% NaCl) by ID injection into the ear. After 120 h of the treatment, immune cells isolate from the treated ear (A-E) and skin-dLN (F) were analyzed by flow cytometry. (A) Total number of $CD3^+$, $CD220^+$, Gr-1^+, and $CD11b^+$ cells in the population gated on $CD45^+$ cells. (B) Total number of $CD4^+$ and $CD8^+$T cells in the population gated on $CD45^+CD3^+$cells. (C) Total number of Th1, Th2 and Th17 cells. The cells were gated on $CD45^+CD3^+CD4^+$, then IFN-γ^+, IL-4^+ and IL-$17A^+$ cells were detected as Th1, Th2, and Th17, respectively. (D) Total number of Tc1, Tc2, and Tc17 cells. The cells were gated on $CD45^+CD3^+CD8^+$, then IFN-γ^+, IL-4^+ and IL-$17A^+$ cells were detected as Tc1, Tc2, and Tc17, respectively. (E) Total number of γδT, Treg, and NKT cell. The cells were gated on $CD45^+CD3^+$, then TCR$\gamma\delta^+$, $CD4^+CD25^+Foxp3^+$, and CD1d-Tetramer (α-GarCer loaded)$^+$ cells were detected as γδT, Treg, and NKT cell, respectively. (F) Total number of Th1 and Tc1 cells in dLN. Cells were gated on $CD3^+CD4^+$ or $CD8^+$, then IFN-γ^+ were detected as Tc1 and Tc2, respectively. In each experiment, 3-5 mice were used for the vehicle and SA-LP ID injection. The isolated cells from the ear and dLN were pooled, and used for flow cytometry analysis. Data are shown as the mean and SD of at least three samples of a single experiment and are representative of at least three independent experiments. The Student's *t*-test was used to analyze data for significant differences. Values of * $p < 0.05$, ** $p < 0.01$ and *** $p < 0.001$ were regarded as significant.

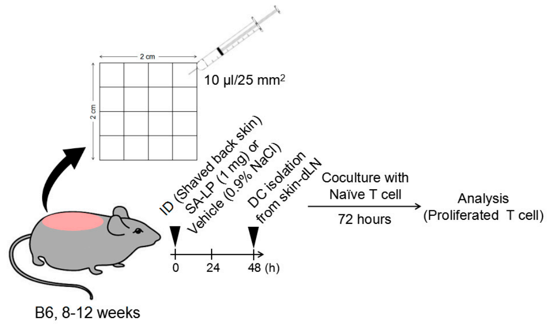

Figure S5. In vitro antigen presentation for naive T cell. WT mice were treated with SA-LP (1 mg) or vehicle solution (0.9% NaCl) by ID injection into the shaved back skin. After 48 h of the treatment, the cells were extracted from skin-dLNs, and DCs were isolated from the pooled cells. The isolated DCs were cocultured with splenic naive T cells for 72 h. The proliferated cells were analyzed by flow cytometry.

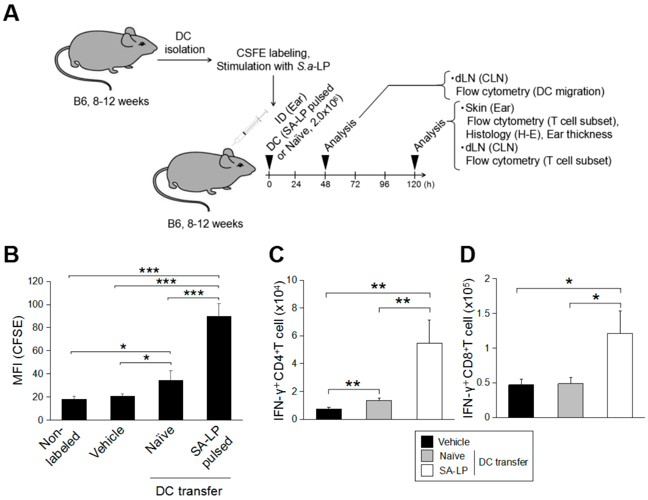

Figure S6. SA-LP activated DC transfer induces inflammation on the skin. (A) Design of the DC transfer. Primary DCs were isolated from WT mice, then the DCs were treated with SA-LP (10 µg) for activation, and labeled with CFSE. The activated or naive DCs (2.0 x 10^6) were ID injected into the ear. After 48 and 120 h, the ear and skin-dLN were used for analysis. (B) The migration of transferred DC into skin-dLN. After 48 h of the treatment, the cells isolated from skin-dLN were analyzed by flow cytometry. $CFSE^+$ cells in mDCs ($MHCII^{hi}CD11c+$) were distinguished as original transferred DCs. (C-D) The number of Th1 and Tc1 cells in skin-dLN of DC transferred mice. After 120 h of the treatment, the cells isolated from skin-dLN were analyzed by flow cytometry. The cells were gated on $CD3^+CD4^+$ or $CD8^+$, then IFN-γ^+ cells were detected as Tc1 (C) and Tc2 (D), respectively. Data are shown as the mean and SD of at least three samples of a single experiment and are representative of at least three independent experiments. The Student's *t*-test was used to analyze data for significant differences. Values of $*p < 0.05$, $**p < 0.01$ and $***p < 0.001$ were regarded as significant.

© 2018 by the authors. Licensee MDPI, Basel, Switzerland. This article is an open access article distributed under the terms and conditions of the Creative Commons Attribution (CC BY) license (http://creativecommons.org/licenses/by/4.0/).
